# Durability of smartphones: A technical analysis of reliability and repairability aspects

**DOI:** 10.1016/j.jclepro.2020.125388

**Published:** 2021-03-01

**Authors:** Mauro Cordella, Felice Alfieri, Christian Clemm, Anton Berwald

**Affiliations:** a European Commission, Joint Research Centre (JRC), Directorate B – Growth and Innovation, Circular Economy and Industrial Leadership Unit, Edificio EXPO – C/Inca Garcilaso 3, 41092, Seville, Spain; b Fraunhofer Institute for Reliability and Microintegration IZM, Dpt. Environmental and Reliability Engineering, Gustav-Meyer-Allee 25, 13355, Berlin, Germany

**Keywords:** Circular economy, Durability, Reliability, Repairability, Smartphones, Trade-offs

## Abstract

Smartphones are available on the market with a variety of design characteristics and purchase prices. Recent trends show that their replacement cycle has become on average shorter than two years, which comes with environmental impacts that could be mitigated through a prolonged use of such devices. This paper analyses limiting states and design trends affecting the durability of smartphones, and identifies reliability and repairability measures to extend the product lifetime. Technical trade-offs between reliability and repairability aspects are also discussed.

Smartphones are often replaced prematurely because of socio-economic and technical reasons. Specific hardware parts (e.g. display, battery, back cover), as well as software, can be critical. Improving the reliability of smartphones can reduce the occurrence of early replacements. Apart from the bottom-line consideration of reliability aspects for electronics, this can be pursued through the design of devices which: i) are resistant to mechanical stresses; ii) implement durable batteries; iii) offer sufficient adaptability to future conditions of use (e.g. in terms of software/firmware updates, memory and storage capacity). However, if and when failures occur, repairs have to be rapid and economically viable. This can be facilitated through modular design concepts, ease of disassembly of key parts, availability of spare parts and repair services. As common elements of the two strategies, easily-available instructions on use, maintenance and repair are also needed.

The analysis of devices on the market suggests that it is possible to design satisfactorily reliable devices without compromising repairability excessively. However, trade-offs between these two aspects can occur. Considerations about reliability and/or repairability should be integrated in the design of all smartphones.

The findings of this paper can be used by decision makers (e.g. manufacturers, designers, consumers and policy makers) interested in enhancing the durability of smartphones. This is particularly timely considering the policy attention on smartphones at the EU level.

## Highlights

• Limiting states and design trends affecting durability of smartphones are analysed.
• Early replacement reasons can be due to technical failures (e.g. display, battery).
• Measures to increase reliability and repairability are identified and discussed.
• Trade-offs between reliability and repairability are highlighted.
• Findings can be used by decision makers to improve the durability of smartphones.

## 1 Introduction

Smartphones were created in the late 1990s, and they have grown on the global market since 2007 to become a very popular tech device, with an estimated 1.7 billion units sold worldwide in 2020 (Statista, 2018a,b). Smartphones are available with a variety of models and characteristics, and can be classified into low-end, mid-range and high-end devices on the base of their selling price (see Table 1).

**Table 1:** Classification of smartphone devices based on their selling price (Statista, 2018c).

Category	Price (USD)	Market share
Low-end	<150	26–34%
Mid-range	150–550	40–50%
High-end	>550	20–28%
Average		
- 2010	440	
- 2015	305	

The manufacturing and use of smartphones come with environmental impacts (Suckling and Lee, 2015) such as consumption of critical raw materials and emissions of greenhouse gases (GHG) (Manhart et al., 2016). In particular, the contribution of smartphones to the global GHG emissions derived from the Information and Communication Technologies (ICT) sector is increasing so fast that it could exceed GHG derived from desktops, laptops and displays (Belkhir and Elmeligi, 2018).

The “European Green Deal” (European Commission, 2019) and the new Circular Economy Action Plan (European Commission, 2020) of the European Commission highlight the importance of implementing measures to improve the material efficiency of the ICT sector. By analogy with the definition of energy efficiency given by the European Commission (2012), Cordella et al. (2020a) defines material efficiency as indicated in Eq. (1):

(1) Material efficiency = Output of a system / Input of materials

Where:

• Output refers to the performance of a system (e.g. in terms of functions, services or goods delivered);
• Input refers to the materials required to deliver that output.

The material efficiency of a product can be improved through approaches aimed at promoting an overall reduction of material consumption, waste production and environmental impacts (Allwood et al., 2011; Huysman et al., 2015). These approaches include, among others, extending the lifetimes of products (e.g. through measures addressing durability, maintenance, repair and reuse of products) and recovering materials through recycling processes.

The relevance of such approaches for mitigating the environmental impacts of a product depends on the magnitudes of impacts associated with its different life cycle stages (Iraldo et al., 2017; Sanfelix et al., 2019; Tecchio et al., 2016), which are in turn influenced by specific design characteristics of the product, and user behaviour. Material efficiency is very important in the case of smartphones since materials, parts (in particular electronics and display) and manufacturing of the device shape the life cycle impacts of the product to a large extent (Cordella et al., 2020b). To address energy and resource efficiency concerns, an Ecodesign and Energy Labelling preparatory study on mobile phones, smartphones and tablets, with a particular focus on material efficiency aspects, was launched by the European Commission (Ecosmartphones, 2020).

Increasing the durability of smartphones is an interesting option to reduce the environmental impacts of these devices (Cordella et al., 2020b). From an engineering perspective, this can be pursued by improving the reliability of the device (i.e. reducing the likelihood of failures) and/or its repairability (i.e. facilitating its restoring in case of failure). Both reliability and repairability seem to be appealing characteristics for consumers, although there might be a slight consumer preference for reliability (Cerulli-Harms et al., 2018). Furthermore, both strategies can yield environmental benefits (Cordella et al., 2020b). Benefits could be potentially higher for more reliable smartphones, where impacts associated with the repair or replacement of the device are avoided, or at least delayed. Higher collection rates of devices that are no longer in use can lead to additional environmental benefits associated with the recovery of value from product, parts and materials (Andrae, 2018; Cordella et al., 2020b).

Material efficiency strategies should not be seen as a pool of separate alternatives, but rather as a set of interconnected options that may mutually affect one another (Cordella et al., 2019). A critical analysis of how one strategy can technically affect the other would enable decision makers (e.g. designers, consumers, regulators) to take informed decisions.

**Fig. 1:**
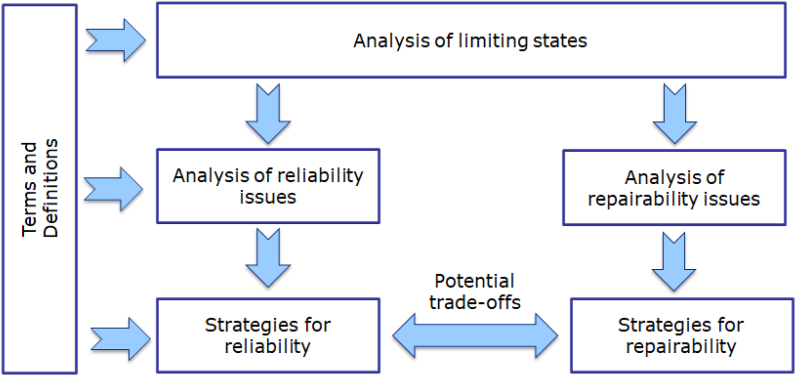
Approach followed for the analysis of reliability and repairability of smartphones.

Building on information available in the literature for smartphones, this paper aims to analyse limiting states affecting the durability of these devices, identify main design trends and options to improve the reliability and repairability of smartphones, as well as to discuss technical trade-offs between these two strategies. The approach followed is shown in Fig. 1 and described in Section 2. Results are presented and discussed in Section 3. Practical applications of the paper and future research orientations are addressed in the conclusions.

## 2 Materials and methods

### 2.1 Terms and definitions

In response to Mandate 543 (European Commission, 2015), the European Standardisation Organisations set up a technical committee (CEN/CENELEC JTC10) that worked out general standard methods for the assessment of material efficiency aspects of energy-related products, such as durability (EN 45552:2020), the ability to repair, reuse and upgrade energy-related products (EN 45554:2020), as well as a series of terms and definitions related to material efficiency (prEN 45550:2018). These standards have been used as the basis for the definition of material efficiency terminologies used in this paper, as summarised in Table 2.

**Table 2:** Definitions of key terms used in this paper.

Term	Definition
Durability	Ability to function as required, under defined conditions of use, maintenance and repair, until a limiting state is reached
Functional lifetime	Time a product is used until the requirements of the user are no longer met, due to the economics of operation, maintenance and repair or obsolescence
Limiting event	Occurrence, which results in a primary or secondary function no longer being delivered
Limiting state	Condition after one or more limiting event(s)
Reliability	Probability that a product functions as required under given conditions, including maintenance, for a given duration without limiting event
Repair	Process of returning a faulty product to a condition where it can fulfil its intended use
Reuse	Process by which a product or its parts, having reached the end of their first use, are used for the same purpose for which they were conceived
Technical lifetime	Time span or number of usage cycles for which a product is considered to function as required, under defined conditions of use, until a failure occurs
Technical withstand (or resistance)	Capability to withstand stresses such as, for example, electrical, mechanical and environmental stresses
Upgrade	Process of enhancing the functionality, performance, capacity or aesthetic of a product

**Fig. 2:**
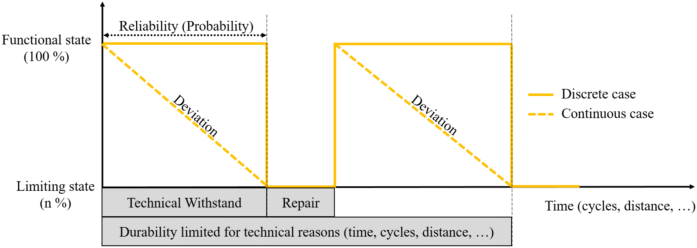
Relationship between reliability, repair and durability (adapted from EN 45552:2020).

The durability of a product can be limited for technical reasons (e.g. time, cycles, distance) and depends on the resistance of the product to loads and degradation mechanisms (reliability), and the ability to bring it back to a functional state (through repair) once a limiting state is reached. Fig. 2 illustrates the relationship between reliability, repair and durability. A product works (functional state) until a limiting event occurs and required functions are no longer delivered. The product is then in a limiting state. It is transitioned back into a functional state if repair actions are applied. Two cases are identified depending whether the functional state immediately drops, or progressively degrades (e.g. in the case of batteries) to a limiting state (discrete or continuous case, respectively).

The durability of a product affects its technical lifetime and, when maintenance and repair aspects (including software issues) are considered, its functional lifetime (European Environmental Agency, 2017). Psychological aspects (perceived obsolescence) can also affect the actual longevity (useful lifetime) of a product (Makov and Fitzpatrick, 2019), although this is not the focus of this paper.

### 2.2 Analysis of limiting states

A product can function as required, under defined conditions of use, maintenance and repair, until a limiting state is reached, i.e. when one or more required functions/sub-functions are no longer delivered (Alfieri et al., 2018). As a preliminary step, typical limiting states of smartphones are analysed based on information about:

1. Length of replacement cycles and reasons for replacing devices;
2. Frequencies and causes of failure and repair/upgrade operations;
3. Barriers hindering the lifetime and repair of devices.

The aim of the analysis is to identify impacted functions and parts, to understand where options to improve the reliability and repairability of smartphones could yield more tangible results.

The information necessary for the analysis presented in this paper was collected from technical and policy research in the field (Bakker et al., 2014; Prakash et al., 2020; Watson et al., 2017), behavioural surveys (Counterpoint Research, 2016; Kantar World Panel, 2017), as well as from organisations specialised in product design (Bullitt Group Research, 2017), product testing (OCU, 2017, 2018, 2019) and repair (Click Repair, 2019; Phonerepaircompare, 2020).

### 2.3 Reliability and repairability analysis

Two design strategies with which to promote an extension of the functional lifetime of smartphones are analysed:

1. Improving the device reliability;
2. Improving the device repairability.

Measures for improving the reliability of the device are identified through a “reliability analysis”, aimed at analysing stress conditions, technical aspects and misuses that could cause failures and loss of functions, as well as understanding how limiting states could be potentially delayed.

Measures for improving the repairability of the device are identified through a “repairability analysis”, aimed at understanding how to fix technical problems and facilitating the restoring of a functional state.

Considerations about the upgradability of smartphones for future conditions of use (Cordella et al., 2020b) are also integrated in the analysis.

General reliability and repairability aspects for smartphones were gathered from technical studies analysing efficiency aspects and circular business models for smartphones (Andrae et al., 2020; Cordella et al., 2020b; Flipsen et al., 2019; Watson et al., 2017).

This evidence was coupled with information for key parts from the technical literature on smartphones (e.g. Clemm et al. (2020a) and Schischke et al. (2016, 2017) for the display and back cover; Andrae (2018), Clemm et al. (2016, 2020b), Groot (2014), Proske et al. (2020) and Vetter et al. (2005) for the battery), as well as from organisation specialised in the design (Bullitt Group Research, 2017) and repair of smartphones (iFixit, 2019; Wertgarantie, 2017). Possible testing methods to support the assessment and verification of such measures (e.g. TCO Certified, 2018; Vanegas et al., 2018) were also identified.

An analysis of main market trends for key design aspects relating to display, back cover and battery of smartphones was also carried out (Berwald et al., 2020). For this analysis, market data was retrieved from Counterpoint Research (2020), comprising sales volumes and market shares (in units) for the best-selling smartphone models in Europe from 2010 to 2019. The data lists a varying of smartphone models with the highest market share for each year; 16 models for 2010, 20 for 2011 and 2012, 22 for 2013, and 25 for 2014–2019, covering between 41% and 72% of the entire smartphone market. The market data was then complemented by data pertaining to specific design aspects of the smartphone models, also contained in the market data.

The following design aspects were addressed: the use of glass as back cover material, Ingress Protection (IP) codes, embedded batteries, use of joining and fastening techniques. Thereby, the market-weighted development of those design aspects associated with material efficiency could be analysed over the past decade.

Compatibility and possible conflicts between reliability and repairability measures were addressed, also based on the available information and on the analysis of the repairability scores by iFixit for 100 smartphone devices (iFixit, 2019; Cordella et al., 2020b) and the corresponding IP67/68 codes (GSMArena, 2019).

## 3 Results

### 3.1 Limiting states

Globally, the replacement cycle of smartphones was about 21 months in 2016, with apparently longer cycles in developed markets (Counterpoint Research, 2016; Kantar World Panel, 2017). Interestingly, the average replacement cycle is close to the smartphone service contract length typically signed by consumers in Germany (Prakash et al., 2020), as well as in other countries. As a comparison, it is calculated that the median lifespan of a (pre-smartphone era) mobile phone decreased from 4.8 to 4.6 years between 2000 and 2005 (Bakker et al., 2014).

**Fig. 3:**
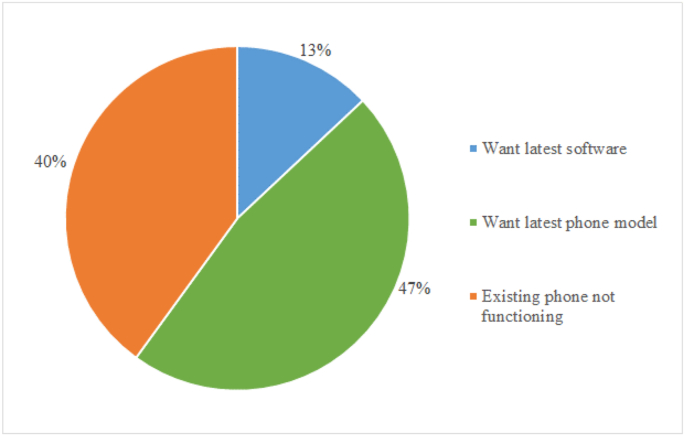
Reasons for smartphone replacement (Watson et al., 2017).

The decision to replace a smartphone is often based on a perception of functional obsolescence driven by new models on the market (Prakash et al., 2020). However, other important causes of replacement are technical obsolescence due to loss of performance, failures and breakages, as well as functional obsolescence due to the lack of software support (Watson et al., 2017), as Fig. 3 shows.

The results of a consumer survey published by OCU (2018) suggest that frequency of failures in smartphones can be relatively high compared to other products. 47% of failures were reported to occur in the first two years of use, while other 39% of failures occurred between the second and third year. The highest number of problems was related to battery (42%) and operating system (OS) (14%). Additional survey results (OCU, 2019) show that consumers are dissatisfied with failures occurring in batteries and touch screens, although this can vary from brand to brand.

Dropping a smartphone on a hard surface (43%) and its coming into contact with water (35%) are other important failure causes (Watson et al., 2017). Such product failures are often due to product misuse by consumers and are therefore not covered by a legal guarantee. It was reported that 34% of EU consumers experienced damage to their devices in a three-year observation period (Bullitt Group Research, 2017).

**Fig. 4:**
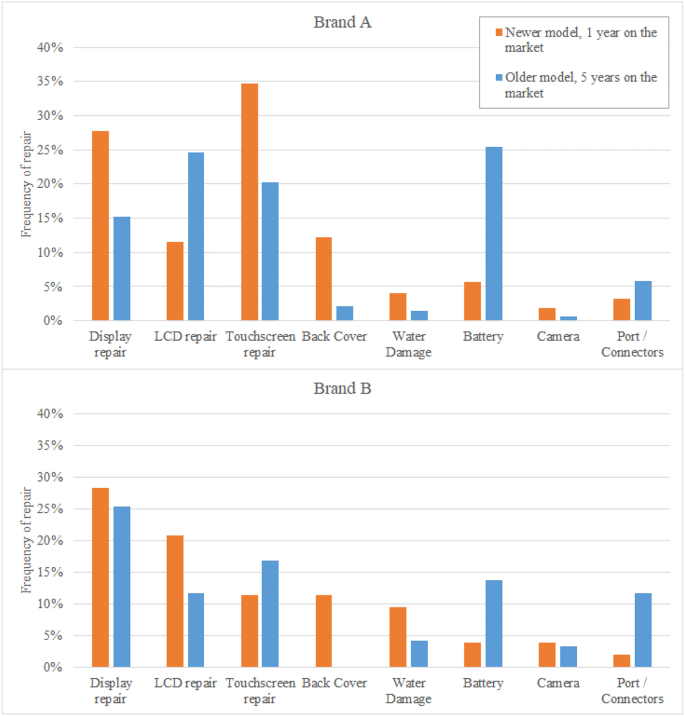
Frequency of common repair requests for two popular brands of smartphones (source: Phonerepaircompare (2020), data query: May 13, 2020).

Apart from failure frequencies, statistics on repair requests can also provide indications about limiting states, as well as technical barriers to repair. Fig. 4 reports repair requests in the UK for older and newer generations of smartphone models produced by two brands (Phonerepaircompare, 2020). For older and newer models of both brands, a major portion of repair requests was associated with problems related to the display and its components.

Other repair organisations (Click Repair, 2019) confirm that damage to displays for smartphones are the most frequent failures leading to limiting states. Interestingly, Fig. 4 shows that the repair (replacement) of back covers has become more frequent for more recent models. Display and back cover repairs are often linked to cracks or scratches that can appear already in the first year of use. Later, new categories of repair requests appear that are related to aging mechanism (e.g. battery/ports) (Prakash et al., 2020). At first glance, repair requests data in Fig. 4 seem to indicate that battery replacements have dropped with newer generations of smartphones. However, this is misleading, since the monitoring period for newer models was 1 year, compared to 5 years for the older models, while the occurrence of battery issues has been historically more probable after use periods of 2–3 years.

Other important factors that can affect the functional lifetime of smartphones are related to the software/firmware supporting period offered by manufacturers. Although the absence of OS updates does not generate any immediate failure, it can result in a loss of security and, in the long term, it could affect the functionality of the installed applications (OCU, 2017). Furthermore, the lack of sufficient memory capacity in the device could create problems for the installation of software and firmware updates, thus making the use of smartphone difficult. Similarly, other components that define the computational performance of smartphones, particularly the main processor (system on chip, SoC) and the available amount of working memory (random access memory, RAM), may limit the technical feasibility of a smartphone to be compatible with the higher performance requirements of software/firmware updates.

### 3.2 Reliability analysis

#### 3.2.1 General considerations for smartphones

Stress factors can be linked to environmental (e.g. ambient temperature and humidity) or operating conditions (e.g. electrical stresses, mechanical shocks and vibrations, ingress of dust and water), and can be influenced by several aspects such as the materials used, and the dimension and shape of parts. The main failures and degradation mechanisms that may cause limiting states for smartphones are outlined in Table 3 (Cordella et al., 2020b).

**Table 3:** Main failures of smartphones, affected parts and failure and degradation mechanisms.

Part	Main failures	Failure and degradation mechanisms
Display	Display glass cracked, scratched, splintered	Accidental drops or other mechanical stresses (shocks, vibrations)
	LCD failure: black screen, broken/dead pixels, no background light	
	The touchscreen does not respond as expected	
Back cover	Breakage (e.g. cracks, scratches)	Accidental drops or other mechanical stresses (shocks, vibrations)
Battery	Loss of performance	Aging of the battery
	Battery not charging	External power supply/charging port/battery connection failure
	Overheating	Increased internal resistance
Operating System	Malfunctioning/loss of security and performance	Software, OS and/or security updates not available Lack of sufficient capacity (memory) or performance (low RAM, outdated SoC)
Electronics	Short circuits, disconnection of parts	Stress conditions (e.g. water and dust exposure, shocks, vibration)

In general, limiting states of smartphones could be avoided or postponed through:

• The design of more robust devices, the use of high-quality parts and the application by users of protective accessories (such as screen protectors and external cases);
• Future-proofing devices offering sufficient memory capacity and performance (RAM, SoC), as well as the availability of software/firmware and security updates over time;
• The provision of information about the correct use and maintenance of the device.

Furthermore, the design of more robust devices might be promoted via legal guarantees and/or extended guarantees, including failures due to accidental drops and contact with water. However, as a side effect, it cannot be excluded that this could potentially lead to consumers taking less care of their devices.

Additional reliability considerations specifically addressing key parts of smartphones are provided in the following sections 3.2.2, 3.2.3.

#### 3.2.2 Display and back cover

**Fig. 5:**
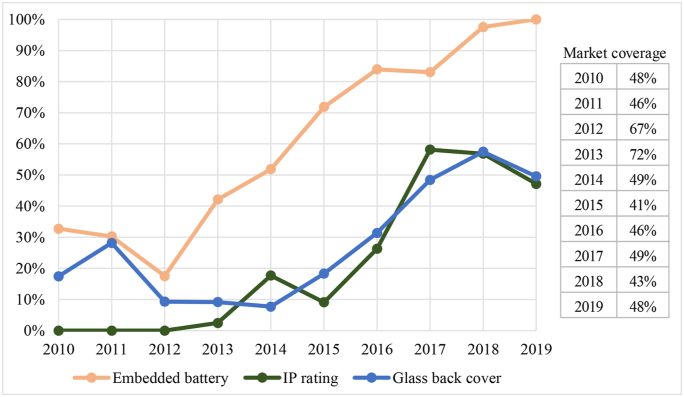
Evolution of design aspects with implications on reliability for best-selling smartphones in Europe (Berwald et al., 2020).

Most modern smartphones feature a chemically strengthened glass display and more and more devices also come with glass on the back (Fig. 5).

Glass is relatively more scratch-resistant than commonly used plastics, it ensures better signal reception (Wi-Fi, LTE, Bluetooth) than metal, and it does not interfere with wireless charging (unlike metals). The main disadvantage is the fragility of the material. Although glass panels are becoming more resistant, displays are still affected by a relatively high frequency of failures. Almost 75% of all display damages are due to drops on corners or edges (Schischke et al., 2017).

The smartphone market has steadily moved towards larger display sizes (from an average of 3.2 inch in 2010 to 6.0 inch in 2019, among the best-selling smartphones in Europe) and smaller bezels (from an average screen-to-body ratio of 46% in 2010 to 80% in 2019) (Clemm et al., 2020a). All-glass, bezel-free smartphones could increase the area of the phone that is susceptible to cracks and breakages.

In recent years, foldable OLED displays and clamshell foldable smartphones entered the market. The durability of foldable displays has not been comprehensively assessed in the literature; however, concerns exist regarding the longevity of the flexible panels, the hinges, and the material covering the displays (BBC, 2020).

Smartphones can be designed to withstand display stresses due to drops on a hard surface, contact with water and ingress of dust (Watson et al., 2017). The following test methods are applied by manufacturers and consumer organisations to measure the resistance of devices to such stresses:

• Drop tests (e.g. based on IEC 60068-2-31:2008 or Method 516.7 of the MIL-STD-810H) and functional requirements to pass the tests (e.g. undamaged display, intact back cover, device resuming and functioning normally according to TCO Certified (2018));
• Display scratch resistance tests (e.g. based on ISO 1518-1, 2019, ISO 1518-2, 2019);
• Water and dust proof tests (IEC 60529).

IEC 60529 is used to classify products based on an Ingress Protection (IP) code that gives information about the degree of protection against the intrusion of solid objects, dust, and water in electrical enclosures. The IP code consists of two digits: the first digit refers to solid particle IP, with 6 meaning complete protection against dust. The second digit refers to liquid IP, with 7 or 8 meaning that protection against immersion was tested (up to or deeper than 1 m, respectively).

The first market-relevant smartphones with IP code entered the market in 2013 with a share of 2%, which increased to 58% in 2017 (Fig. 5). The apparent decline in 2018 and 2019 stems from a class of mid-range devices with a high market share, but lack of IP code.

Rugged smartphones are a niche market and are commonly completely sealed within a thick housing to protect them against damage from, inter alia, water, shock, dust, and vibration. They comply with IP67/68 codes and are drop tested onto a hard surface from a minimum of 1.2 m (Bullitt Group Research, 2017). Some rugged smartphones are labelled as compliant with the MIL-STD-810, which provides general guidelines and laboratory test methods for different world climatic regions.

Complementarily to the considerations above, screen protectors and external cases can reduce the occurrence of limiting states caused by drops or other mechanical stresses. For example, the frequency of brakeage could be halved when a protective external case is applied (Wertgarantie, 2017).

#### 3.2.3 Battery

All rechargeable batteries degrade over time and with use, resulting in a loss of available capacity, energy and/or an increase in impedance, and therefore a reduction in power and efficiency. The performance (autonomy between charges) and lifetime of batteries are very important aspects for smartphones (Benton et al., 2015) since customers demand highly portable devices with batteries that last a full day. A long battery lifetime can delay the replacing of this part and the entire device (Andrae, 2018).

The lifetime of batteries is measured in two ways:

1. Calendar life: time during which the battery can be stored, without or with only minimal use, before its capacity permanently decreases below e.g. 80% of the initial capacity, and
2. Cycle life: number of times (cycles) a battery can be fully charged and discharged before it becomes unsuitable for a given application, e.g. when it can only be charged up to 80% of initial capacity.

A long calendar life and high cycle life are desirable characteristics of batteries. Both aspects can be evaluated in laboratory tests to provide insights into the expected lifetime of tested batteries in the field. Commonly, the battery endurance in cycles is tested as an indicator for the battery quality and expected lifetime.

The battery endurance in cycles can be tested according to IEC EN 61960-3 as:

a. Remaining full charge capacity of the battery compared to its initial capacity, after a specified number of charge/discharge cycles (e.g. 300, 500, or 1000), or
b. Minimum number of full charge/discharge cycles a battery can undergo while retaining a specified share of its initial capacity (e.g. 80% or 60%).

Some manufacturers make statements about the expected lifetime of their batteries (e.g. retaining up to 80% of the original capacity after 500 full cycles). Batteries used in a smartphone released in 2019 were reported to endure more than 850 full charge/discharge cycles on average before their capacity dropped below 80% (Proske et al., 2020). This translates into a lifetime of the battery of two to three years, when an average intensity of use is assumed (Clemm et al., 2016). It should be noted that batteries may be used at below 80% capacity; however, a more rapid fade may occur due to common ageing mechanisms within the cell.

Practically all devices among the best-selling smartphones now feature embedded batteries, which cannot be easily removed without the use of tools (see Fig. 5). Cordella et al. (2020b) reported that embedded batteries can require less materials to protect the battery cells. In fact, the housing would provide the mechanical containment of the cells, so that the housing of the battery itself can be reduced to semi-flexible foils rather than solid metal or plastic housing. Furthermore, the battery electronics may be integrated into the existing system circuity, resulting in an overall higher volumetric and gravimetric energy densities.

Comprehensive insights into dominant ageing mechanisms of lithium-ion (Li-ion) batteries were provided by Groot (2014) and Vetter et al. (2005), such as: losses of active and accessible electrode material and active lithium ions, loss of conductivity in the electrodes or the electrolyte, and decomposition of the electrolyte. Factors influencing the rate of capacity fade in Li-ion batteries are temperature, charge/discharge cycling, average cell voltage level, and charge and discharge rate, among others. While the ambient temperature can be addressed by manufacturers with thermal design, the charging rate and the average cell voltage level can be managed by smart battery management software. For instance, higher charging rates have been shown to lead to accelerated ageing of smartphone batteries (Clemm et al., 2020b). Further, high average cell voltage may be limited with smart charging algorithms that cap the charging process before it reaches 100%, and only fully charge the device when needed by the user. The provision of information about the correct use of lithium-ion batteries can also contribute to extend the battery life.

Smartphone users are generally not able to verify the cycle endurance of their batteries in practice, as most manufacturers do not employ systems that track and report the number of charge cycles or the state of health. However, such data may be useful for users to better understand the health status of their battery, to verify expected lifetimes, and to estimate the feasibility for a continued use of an aged battery.

### 3.3 Repairability analysis

#### 3.3.1 General considerations for smartphones

Several technical aspects can hinder or facilitate the repair of smartphones (Andrae et al., 2020; Cordella et al., 2019; Flipsen et al., 2019), and by extension its reuse. First, a lack of spare parts and software/firmware updates, as well as their relative costs, can be an important barrier to repair. According to Watson et al. (2017), repairing smartphones could be worthwhile even 3–5 years after sale. However, there is huge variation in the cost of repair depending on involved parts, design choices, labour costs and socio-economic context. Repairing the display could cost up to 15–40% of the purchase price of new devices, while other repair operations could cost about 10% of the purchase price or more (Cordella et al., 2020b). Repair could be easier if standardised interfaces were used (e.g. for connectors and External Power Supplies (EPS)) and supported by data transfer/deletion and password reset and restoration embedded functions. On the other hand, sub-standard quality of spare parts and repair operations may undermine the functionality of the device and the brand reputation (Cordella et al., 2020b).

Secondly, another important aspect is the sharing (e.g. via the web) of maintenance and repair information, such as the availability and cost of spare parts and repair services in different countries. However, some information could be restricted to a subset of professional and/or authorised repairers because of safety, liability and/or confidentiality issues (Cordella et al., 2019).

**Fig. 6:**
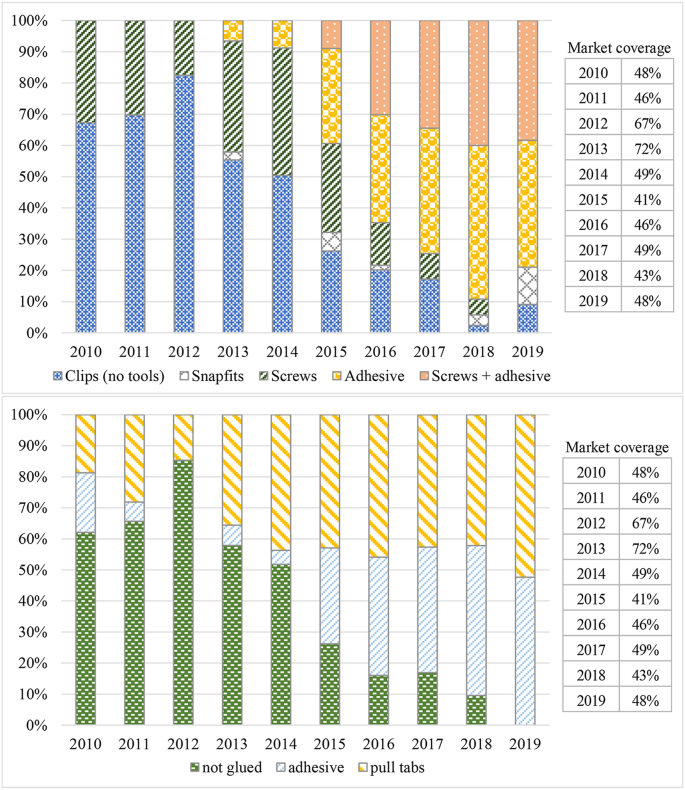
Evolution of joining and fastening techniques applied to housing (above) and batteries (below) for best-selling smartphones in Europe (Berwald et al., 2020).

Thirdly, currently a technical and economic barrier to repair is the difficulty to disassemble and reassemble parts of smartphones (batteries and displays). Product disassembly and reassembly can be eased by adopting a modular design and/or using removable and reusable fasteners. Furthermore, do-it-yourself (DIY) repair can be facilitated if repairs can be carried out with basic or commercially available tools (e.g. suction cup pliers, spudgers) (Flipsen et al., 2019). However, the trend is towards highly integrated, thin and light smartphones, also in response to a demand by customers of highly portable and durable devices (see Section 3.2.3). In many cases this meant an increased use of adhesives in place of joining techniques that can be separated without the use of tools (see Fig. 6).

iFixit (2019) created a global support platform for the repair of smartphones. This platform provides instructions for the replacement of batteries, displays and other parts, as well as repairability scores for a number of models. Cordella et al. (2020b) analysed the repairability scores calculated by iFixit for 100 devices. The bottom 10% of rated devices received a repairability score between 1 and 3 and were characterised by:

• High difficulty to open the back cover and access parts;
• Intensive use of adhesive, making the disassembly more complex;
• Very difficult replacement of the battery.

Metrics to assess the difficulty/ease of disassembly of parts include the disassembly depth and the disassembly time (Cordella et al., 2019), where the term “disassembly depth” is the number of steps required to remove a part from a product. Factors influencing the disassembly process (e.g. disassembly steps, fasteners, tools) can be combined all together through the calculation of disassembly times (Vanegas et al., 2018).

Indications about the steps and time necessary to disassemble parts of smartphones are provided by Cordella et al. (2020b). The analysis of a sample of 19 products found that disassembly steps range from 15 to 45 for the display (median value = 30), from 1 to 103 for the back cover (median value = 14), and from 2 to 46 for the battery (median value = 28). These corresponded to a disassembly time of 1–120 min for the display (median value = 40 min), 1–180 min for the back cover (median value = 53 min), and 1–120 min for the battery (median value = 30 min). This points out how much variability exists in the design characteristics of smartphones on the market, with their commensurate influence on repair operations.

#### 3.3.2 Display and back cover

The trend towards the increased use of adhesives (see Fig. 6) can in general make the disassembly of parts more difficult. In the case of the display, the entire unit must be commonly replaced since the individual components are fused together and come as one piece. Repair could be very challenging for new foldable phones (iFixit, 2019). Modular design approaches can improve the ability to disassemble and reassemble displays and other parts. However, effective modular design concepts should avoid adopting the permanent fixing of display components, such as the front glass or display module (Schischke et al., 2016).

#### 3.3.3 Battery

In recent models, the removal of batteries requires the intervention of an experienced repairer, since manufacturers have increasingly opted to fasten batteries within the phones using adhesives (see Section 3.2.3). Batteries in the best-selling smartphones of 2019 (48% of the European market) were all fastened within the devices using adhesives (see Fig. 6). Of those, more than 50% used pull tabs that lose their adhesive properties when stretched, enabling the removal of adhered components. However, anecdotal evidence suggests that adhesives with stronger adhesive force have also been used; these are very difficult to separate, even when thermal energy and prying force are applied. This is a hurdle, especially when the smartphone design is such that the battery adheres to the display unit (Clemm and Lang, 2019). This means that the lifetime of the battery can be even more of a decisive factor for users when deciding between replacing or continuing to use their current smartphone device.

### 3.4 Reliability and repairability measures

Measures to improve the reliability and repairability of smartphones are summarised in Table 4, with an indication of possible testing methods supporting their effective implementation. In general, these measures aim at eliminating technical barriers (e.g. through the promotion of improved designs) and/or correcting behavioural patterns that could cause the premature replacement of devices (e.g. via the provision of use and maintenance information).

**Table 4:** Possible measures to improve the material efficiency of smartphones.

Measure	Testing
Reliability	
1) Resistance to accidental drops a. Compliance with specifications of standard drop tests b. Availability of screen protectors and protective cases	a. IEC 60068-2-31 or Method 516.7 of the MIL-STD-810H b. Provision of information and visual check
2) Scratch resistance (screen, camera, fingerprint sensor, etc.)	Display scratch resistance tests (e.g. based on ISO 1518-1, 2019, ISO 1518-2, 2019)
3) Protection from water and dust: compliance with the IP67 or IP68 codes	IEC 60529
4) Battery endurance a. Minimum number of cycles with the battery properly functioning b. Pre-installed battery management software for smart charging and provision of state of health data c. Information for the correct use of the battery	a. IEC EN 61960-3 b/c. Provision of information and visual check
5) Operating System, software and firmware a. Availability of update support for 3–5 years and information on impact/reversibility of updates b. Possible use of open source OS or open source Virtual Machine software enabled c. The capacity of the device allows the installation of next OS versions and future functionalities	Provision and check of information
6) Guarantee ^(^∗) a. Inclusion of failures due to accidental drops and contact with water in the legal guarantee b. Extended guarantee	Provision and check of information
Repairability	
1) Disassemblability a. Battery, display and back covers to be removable in less than a defined number of steps/minutes b. Non-removable and non-reusable fasteners (e.g. adhesives) are avoided for the assembling of batteries, screens and back cover c. Battery, display and back cover to be easily accessible and replaceable using commonly available tools	Provision and check of information: - Exploded diagram of the device - Illustration of how parts can be accessed, replaced and reassembled - Indication of tools required and associated difficulty (e.g. disassembly steps/time needed – depending on the approach followed)
2) Provision of maintenance and repair information to final users and professional/independent repairers Note: Information to be available for a minimum time (e.g. 3-5 years); some information may be restricted to a subset of repair actors due to safety, confidentiality and liability issues	Provision and check of information
3) Availability of spare parts a. Spare parts are available for at least 3–5 years and can also include approved-by-manufacturer spare parts produced by third parties b. Parts are listed on-line with their prices and are delivered in 2 working days after having received the order c. Standardised interfaces are used (e.g. for connectors and EPS)	Provision of information and availability checked over time
4) Data management functionalities a. Availability of data transfer and deletion functions b) Availability of password reset and restoration of factory tools	Declaration by manufacturers and check of actual availability

∗ Also relevant for repairability in case of inclusion of “repair-first” clauses (Cordella et al., 2019).

**Fig. 7:**
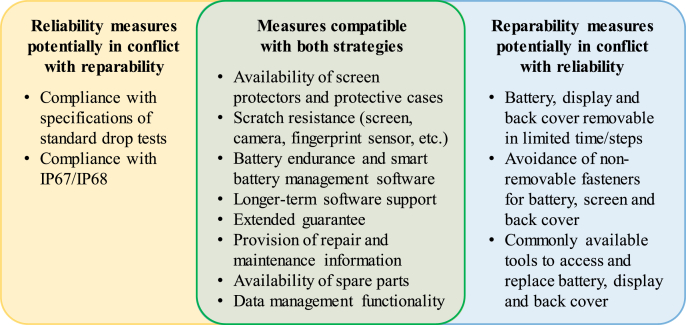
Indication of potential conflicts between reliability and repairability measures, as well as measures that are considered compatible with both strategies.

Measures reported in Table 4 have been classified in Fig. 7 based on their potential influence on reliability and repairability:

• The first group of measures is considered to improve reliability but to present a potential conflict with repairability. This can be the case for complying with drop tests and IP67/68 codes, where more robust design concepts may be necessary (rather than modular designs).
• On the other end of the spectrum, certain measures that may improve repairability could reduce the technical withstand of smartphones. Modular designs aiming at reducing steps and the time to disassemble parts may be in potential conflict with design measures for reliability.
• A third group of measures are considered as being compatible with both design strategies. This includes scratch resistance of glass parts, battery endurance, and longer-term software support, among others.

As stated in the introduction, some manufactures claim to prioritise reliability aspects (e.g. technical withstand and water/dust IP code). This finds support in the trends observed in Fig. 5, where it can be observed that a large share of the best-selling devices has moved towards IP codes (almost 50%) and embedded batteries (100%). Although the latter trend was apparently aimed at designing more powerful devices within a limited volume (see Section 3.2.3), this typically comes with the use of non-removable and non-reusable fasteners (see Fig. 6), which can make the replacement of parts by users, and the repair of the product in general, more difficult.

Cordella et al. (2020b) provides information about iFixit repairability scores and corresponding IP67/68 codes (GSMArena, 2019). Several models with IP67/68 codes received a repairability score between 3 and 7, meaning that it can be possible to design reliable and resistant devices without compromising repairability excessively. This finding is in line with a recent study (Clemm and Lang, 2019) suggesting that some smartphones can achieve good IP codes (IP67) without penalising the ease of replacing batteries. Since then, more smartphones have become available on the market that have both a user-replaceable battery and high IP codes (e.g. rugged devices).

Other manufacturers are focusing on modular design concepts that can improve the repairability and upgradability of smartphones. Such devices can allow users to directly replace modules of smartphones, or even upgrade the functionality of the device. It was pointed out that this could lead to an increase of the material and volume needed to house different parts (Transform Together, 2018). However, smartphones do not necessarily need to be fully modular: hybrid solutions could be feasible and provide material efficiency benefits. Research suggests that to achieve the highest efficiency in terms of material recovery and cost savings, modularity should focus on printed circuit boards (PCBs), display, back cover and battery (Souchet et al., 2017). A challenge still to be solved for modular devices relates to the water and dust IP: to the knowledge of the authors, no modular device has achieved IP67/68 codes.

## 4 Conclusions

This paper analysed limiting states and design trends affecting the durability of smartphones and identified measures to increase the durability of such devices. Trade-offs between reliability and reparability strategies were also analysed.

Smartphones are often replaced prematurely because of socio-economic reasons (e.g. wanting a new model), misuses and technical issues relating to display, battery, back cover, as well as the software. An extension of the product lifetime can be pursued by improving the reliability and repairability of smartphones. The two strategies are often presented as a dichotomy, with apparently a slight preference for reliability over repairability from a design, user and environmental perspective. However, enhancing the repairability of devices can lead to similar benefits and add flexibility (e.g. in case of modular design concepts).

Apart from the bottom-line consideration of reliability aspects for electronics, an extension of the lifetime of smartphones can be achieved through designs that: i) are more resistant to environmental stresses and improper use; ii) use durable batteries; iii) offer sufficient adaptability to future use patterns, functionalities and needs (e.g. in terms of availability of software/firmware updates, memory and storage capacity).

Where failures occur, repair should be rapid and economically viable. Repairability of devices can be enabled by ensuring the availability of spare parts and repair services, and improved via design concepts aimed at facilitating the disassembly of key parts and modules (also in favour of the hardware upgrade).

The analysis of IP codes and iFixit repairability scores of smartphone models suggests that it could be possible to design reliable devices without compromising repairability excessively. However, some technical trade-offs may occur, especially when the product design is focused on one strategy only. This can make difficult to find a “one-size-fits-all” solution. In the absence of strong evidence supporting one strategy over the other, the authors would like to recommend that reliability and/or repairability aspects are systematically integrated in the design of all smartphones.

Furthermore, it is fundamental to act at the consumer level to avoid the premature replacement of old but functioning smartphones. This could be achieved through consumer education activities (as pursued by consumer research and testing organisations), and providing consumers with access to relevant, understandable and reliable information for the use, maintenance and repair of smartphones.

The findings of this paper provide an evidence base that can support designers, consumers and regulators in taking more informed decisions about the manufacture, purchase, use and re-use of smartphone devices. This is particularly timely considering the policy attention on smartphones at EU level.

As future research orientation, the authors recommend undertaking further analysis on how more resistant designs and (extended) warranties could influence user behaviour and durability of smartphones. For example, regulating repair costs within the warranty could be an effective measure to extend the functional lifetime of smartphones (Andrae, 2018). Furthermore, technical considerations should be complemented with a broad sustainable assessment of life cycle impacts associated with alternative material efficiency scenarios (Peña et al., 2020).

The authors would also like to highlight that the public availability of information about reliability and repairability of smartphones (e.g. frequency of failures and repairs) is currently limited. In this work, a retrospective body of evidence was built from different sources, mainly including technical literature for smartphones, surveys from consumer research and testing organisations, as well as the websites of professional organisations. Considering that smartphones are tech devices characterised by fast innovation cycles, the approach presented in this study could be used in the future to follow the evolution of technologies and update data and results. With this aim in mind, it would be desirable to: i) cluster research, technological and industrial competences; and ii) promote open and easy access to such information. In this sense, digitalisation offers important opportunities to facilitate the sharing, extraction and analysis of data, provided that condition (ii) – openness – becomes a reality.

## CRediT authorship contribution statement

**Mauro Cordella:** Conceptualization, Investigation, Writing – original draft, Writing; Coordination. **Felice Alfieri:** Conceptualization, Resources, Investigation, Writing – original draft, Writing. **Christian Clemm:** Conceptualization, Resources, Investigation, Writing – original draft, Writing. **Anton Berwald:** Conceptualization, Resources, Investigation, Writing – original draft, Writing.

## Declaration of competing interest

The authors declare that they have no known competing financial interests or personal relationships that could have appeared to influence the work reported in this paper.
